# Impact of Introducing Intensity Modulated Radiotherapy on Curative Intent Radiotherapy and Survival for Lung Cancer

**DOI:** 10.3389/fonc.2022.835844

**Published:** 2022-05-31

**Authors:** Isabella Fornacon-Wood, Clara Chan, Neil Bayman, Kathryn Banfill, Joanna Coote, Alex Garbett, Margaret Harris, Andrew Hudson, Jason Kennedy, Laura Pemberton, Ahmed Salem, Hamid Sheikh, Philip Whitehurst, David Woolf, Gareth Price, Corinne Faivre-Finn

**Affiliations:** ^1^Division of Cancer Sciences, University of Manchester, Manchester, United Kingdom; ^2^Department of Clinical Oncology, The Christie Hospital NHS Foundation Trust, Manchester, United Kingdom; ^3^Radiotherapy Related Research, The Christie Hospital NHS Foundation Trust, Manchester, United Kingdom; ^4^Christie Medical Physics and Engineering, The Christie Hospital NHS Foundation Trust, Manchester, United Kingdom

**Keywords:** IMRT, lung cancer, radiotherapy, real-world data, big data

## Abstract

**Background:**

Lung cancer survival remains poor. The introduction of Intensity-Modulated Radiotherapy (IMRT) allows treatment of more complex tumours as it improves conformity around the tumour and greater normal tissue sparing. However, there is limited evidence assessing the clinical impact of IMRT. In this study, we evaluated whether the introduction of IMRT had an influence on the proportion of patients treated with curative-intent radiotherapy over time, and whether this had an effect on patient survival.

**Materials and Methods:**

Patients treated with thoracic radiotherapy at our institute between 2005 and 2020 were retrospectively identified and grouped into three time periods: A) 2005-2008 (pre-IMRT), B) 2009-2012 (selective use of IMRT), and C) 2013-2020 (full access to IMRT). Data on performance status (PS), stage, age, gross tumour volume (GTV), planning target volume (PTV) and survival were collected. The proportion of patients treated with a curative dose between these periods was compared. Multivariable survival models were fitted to evaluate the hazard for patients treated in each time period, adjusting for PS, stage, age and tumour volume.

**Results:**

12,499 patients were included in the analysis (n=2675 (A), n=3127 (B), and n=6697 (C)). The proportion of patients treated with curative-intent radiotherapy increased between the 3 time periods, from 38.1% to 50.2% to 65.6% (p<0.001). When stage IV patients were excluded, this increased to 40.1% to 58.1% to 82.9% (p<0.001). This trend was seen across all PS and stages. The GTV size increased across the time periods and PTV size decreased. Patients treated with curative-intent during period C had a survival improvement compared to time period A when adjusting for clinical variables (HR=0.725 (0.632-0.831), p<0.001).

**Conclusion:**

IMRT was associated with to more patients receiving curative-intent radiotherapy. In addition, it facilitated the treatment of larger tumours that historically would have been treated palliatively. Despite treating larger, more complex tumours with curative-intent, a survival benefit was seen for patients treated when full access to IMRT was available (2013-2020). This study highlights the impact of IMRT on thoracic oncology practice, accepting that improved survival may also be attributed to a number of other contributing factors, including improvements in staging, other technological radiotherapy advances and changes to systemic treatment.

## Introduction

Lung cancer is the third most common cancer and the leading cause of cancer death in the UK (1). For some time it has been recognised that better treatments are urgently required to improve lung cancer survival. Over the last two decades, increasing knowledge regarding the biology of lung cancer has led to the development of new systemic agents such as tyrosine kinase inhibitors and immunotherapy, leading to improvements in survival in locally advanced and metastatic non-small cell lung cancer. However outcome of lung cancer patients remains poor compared to the majority of other cancer types (2, 3).

Radiotherapy (RT) plays an important role in the management of lung cancer with over 50% patients receiving this modality at some point during their cancer journey (4). Radiotherapy can either be given with palliative intent to control symptoms, or radically with curative intent – in patients with early and locally advanced disease.

Radiotherapy treatment planning is a careful balancing act between optimal tumour control and limitation of damage to normal tissue. In order to avoid undue toxicity, dose constraints are placed on the normal tissues such as the lungs, heart, oesophagus and spinal cord to minimise functional damage. The radiotherapy dose delivered to the tumour is therefore often limited by the dose that can be safely delivered to the normal tissues. This is particularly challenging in patients who have large volume disease and/or disease close to critical normal structures, such as the spinal cord. In some situations this can lead to patients being treated with a safer, lower, but ultimately palliative dose. As local control correlates with improved survival (5, 6), these patients naturally have a poorer outcome.

Over the last two decades, great advancements have been made in radiotherapy technology (7, 8). Prior to the 1980’s radical lung patients were planned with fluoroscopy, however the introduction of computed tomography (CT) allowed improved tumour localisation and conformal planning. In addition, the advent of the multi-leaf collimator (MLC) enabled fields to be shaped around a target volume. This three-dimensional conformal radiotherapy (3DCRT) has been the gold standard for radical RT to the lung since the 1980’s. Subsequently, 4D planning was introduced which incorporates tumour motion into the radiotherapy planning process, allowing more bespoke plans based on tumour motion and a reduction in margins. In addition there have been improved methods of image guidance, allowing the verification of the tumour position during the treatment course with increasing accuracy. This again has allowed a reduction in tumour margins and therefore dose delivered to normal tissue (9). Despite these improvements in technology, there are still a significant proportion of lung cancer patients, in particular those with locally advanced disease, who are treated with a palliative approach either due to the treatment volume or its proximity to a critical structure (10).

Intensity-modulated radiotherapy (IMRT) is an advanced form of 3DCRT that modifies the intensity of the radiation across each beam, sculpting the high-dose volume around the site of disease and thereby sparing adjacent organs at risk. This technology has been available since the early 2000s, however the routine implementation of IMRT in the setting of lung cancer treatment has been slow, due partly to the increased planning and quality assurance time required by this techniques, and a perceived lack of evidence for using it (11). To date there are a handful of large retrospective studies evaluating 3DCRT against IMRT in lung cancer, and only one publication in a randomised, prospective setting which addresses this issue (12). There is a lack of data on the impact of modern RT technology on patient management and outcome, particularly for patients that are typically excluded from clinical trials (13).

We have been treating lung cancer patients in our institution routinely with IMRT for over a decade. This study aims to evaluate whether the introduction of IMRT has had an influence on the proportion of patients we are able to treat with curative intent over time, and whether this has had any impact on patient survival.

## Methods and Materials

A retrospective review of patients in our institution treated with thoracic RT for lung cancer between 2005-2020 was carried out. Approval was granted to collect and analyse this patient data by the UK Computer Aided Theragnostics (ukCAT) Research Database Management Committee (REC reference: 17/NW/0060).

Patients between 2005-2012 were identified by ICD-10 codes on MOSAIQ and patients between 2013-2020 were identified *via* the Christie web portal (CWP – an in house e-record system designed to collect structured data on patients, tumour characteristics and outcome data). For all patients, data on age, sex, ECOG performance status (PS), stage, gross tumour volume (GTV), planning target volume (PTV) and survival were collected. For patients planned using 4D-CT imaging, GTV data was synthesized from the internal gross tumour volume (iGTV) using a previously published method (14).

Patients were grouped into 3 time periods, determined by the year the first radiotherapy fraction was delivered: A (2005-2008, pre IMRT), B (2009-2012, some availability IMRT) and C (2013-2020, full access IMRT). SABR was introduced in 2011 in our institution. Any patient who received an absolute physical dose of greater than 40Gy was classed as having ‘curative-intent’ thoracic RT. This dose was chosen to cover patients receiving radical doses such as 45Gy/30 fractions twice-daily (EQD2 43.1 Gy) or 40Gy/15 fractions daily (EQD2 42.2 Gy) for limited stage small cell lung cancer (SCLC). For patients receiving palliative radiotherapy, records were manually checked to ensure these patients received palliative radiotherapy to the lung (and not a site of metastatic disease). Those that had not were excluded from this study.

The proportion of patients treated with curative-intent RT was compared between the 3 time periods and the Chi-squared test was used to compare differences between the groups. We performed 2 analyses, one including all stages and the other including only patients with stage I-III. The proportion of patients treated with curative-intent RT was also compared across all PS groupings and stages of disease. For curative-intent patients, the trend of tumour volume treated over time was reviewed and the Mann–Whitney U test used to compare GTV and PTV across time periods. Survival curves were generated using the Kaplan-Meier method and compared using the log-rank test. Univariable and multivariable cox survival models were fitted to evaluate the hazard of being treated in one of the 3 time periods, adjusting for baseline PS, stage at diagnosis, age at the start of treatment and GTV. These analyses were then repeated excluding patients who had received stereotactic radiotherapy (SABR). All statistical analyses were performed in R 4.0.0 (15) with package *survival* v3.1-12 (16).

## Results

In total, 12499 patients were identified as having received radiotherapy to the lung between 2005 and 2020; 2675 in group A (2005-2008, pre IMRT), 3127 in group B (2009-2012, some availability IMRT) and 6697 in group C (2013-2020, full access IMRT). Patients in time period B receiving IMRT were planned with this technique only if 3D conformal radiotherapy was unable to achieve a dosimetrically acceptable radical plan.

Baseline characteristics are presented in **Table 1**. Median age was 70 (63-77), 71 (64-78) and 72 (65-78) in each group respectively. 985 patients received SABR, 0 in group A, 33 in group B and 952 in group C.

**Table 1:** Baseline characteristics.

	A: 2005-2008 n=2675	B: 2009-2012 n=3127	C: 2013-2020 n=6697
Age at start of treatment [median (IQR)]	70.00 [63.00, 77.00]	71.00 [64.00, 78.00]	72.00 [65.00, 78.00]
Sex [n (%)]			
Male	1527 (57.8)	1729 (56.0)	3435 (52.3)
Female	1117 (42.2)	1358 (44.0)	3139 (47.7)
Treatment intent [n (%)]			
Curative	1018 (38.1)	1570 (50.2)	4391 (65.6)
Palliative	1657 (61.9)	1557 (49.8)	2306 (34.4)
SABR [n (%)]	0 (0.0)	33 (1.1)	952 (14.2)
ECOG Performance status [n (%)]			
0	284 (10.6)	281 (9.0)	588 (8.8)
1	852 (31.9)	1071 (34.3)	2301 (34.4)
2	474 (17.7)	762 (24.4)	2012 (30.0)
3	167 (6.2)	348 (11.1)	813 (12.1)
4	3 (0.1)	5 (0.2)	17 (0.3)
Missing	895 (33.5)	660 (21.1)	966 (14.4)
Stage [n (%)]			
I	321 (12.0)	443 (14.2)	1490 (22.2)
II	158 (5.9)	243 (7.8)	628 (9.4)
III	552 (20.6)	810 (25.9)	1875 (28.0)
IV	142 (5.3)	512 (16.4)	1706 (25.5)
Missing	1502 (56.1)	1119 (35.8)	998 (14.9)

SABR, stereotactic ablative radiotherapy; ECOG, Eastern Cooperative Oncology Group.

**Figure 1:**
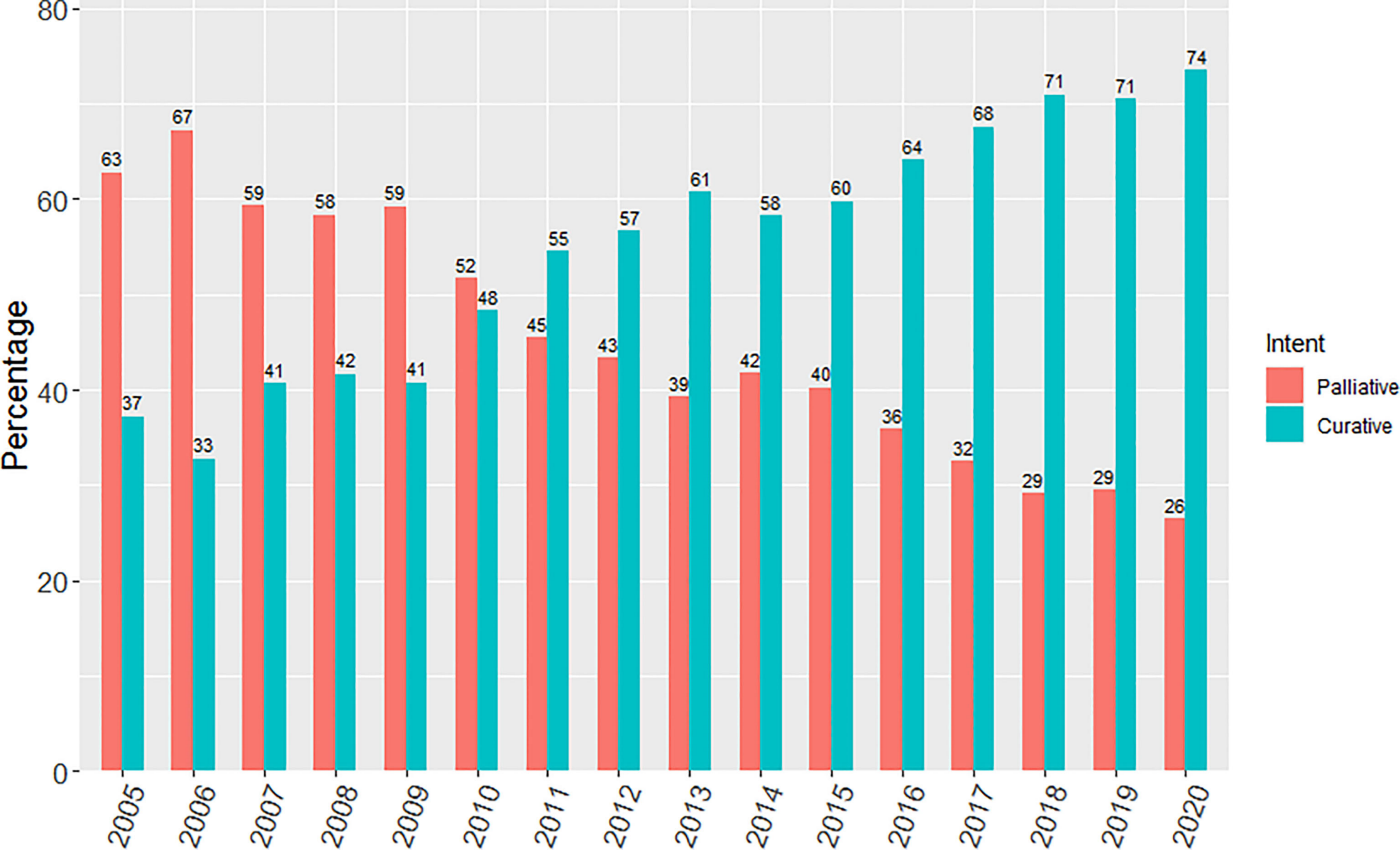
Yearly percentage of patients treated with curative versus palliative intent radiotherapy from 2005 to 2020.

**Figure 2:**
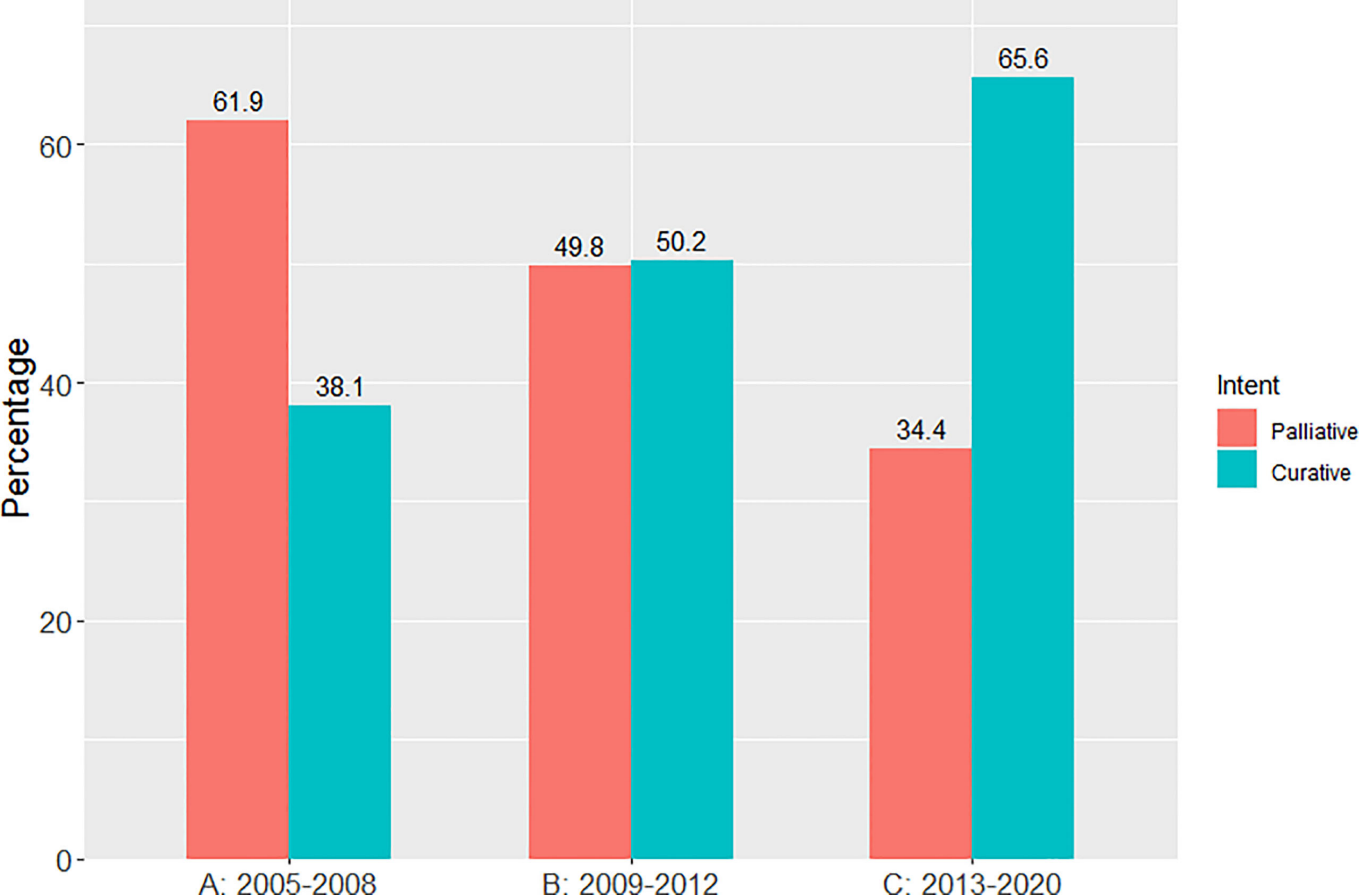
Percentage of patients treated with curative versus palliative intent radiotherapy (whole population) in each of the pre-specified time periods.

**Figure 3:**
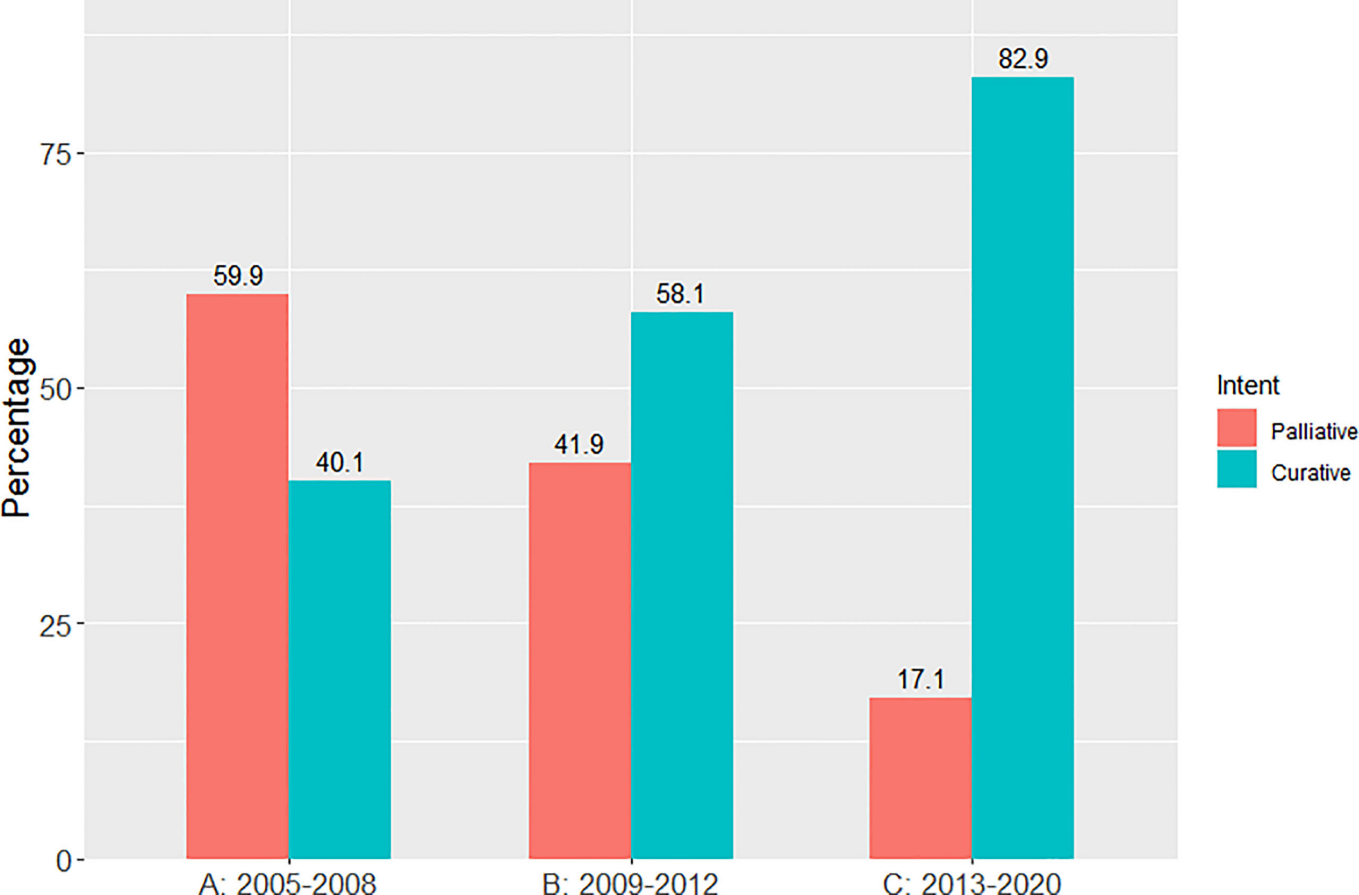
Percentage of patients treated with curative versus palliative intent radiotherapy (stages I-III) in each of the pre-specified time periods.

There was a progressive increase in the proportion of patients receiving curative-intent radiotherapy year on year since 2005, with a step wise change occurring from 2011 as shown in **Figure 1**. This increase in the proportion of patients receiving a curative dose was highlighted further when patients were grouped into the 3 previously specified time periods (**Figure 2**). Patients receiving curative-intent RT increased between groups A (2005-2008) and B (2009-2013) (38.1% to 50.2%, p<0.0001), and B and C (2014-2020) (50.2% to 65.6%, p<0.0001). Results were similar when the patients treated with SABR were removed from the analysis (**Supplementary Figures 1**, **2**). These percentages increased when only stage I-III patients were examined (**Figure 3**) with patients receiving curative-intent RT increasing from 40.1% to 58% to 82.9% in A, B and C respectively.

Further sub-classification according to PS and stage are presented in **Tables 2**, **3** respectively. The proportion of patients treated with curative-intent radiotherapy increased between the three time periods, regardless of PS and stage of disease. Stage IV patients have been included to reflect the increasing use of ‘radical’ radiotherapy to achieve optimal local disease control, typically in the setting of oligometastatic disease. Results were similar when patients treated with SABR were removed from the analysis (**Supplementary Tables 1, 2**). **Table 4** presents sub-classification according to PS for stage III patients only, showing that the proportion of curative-intent patients has increased across all PS for these patients.

**Table 2:** Proportion of patients treated with curative-intent radiotherapy across each PS and time period.

PS	A: 2005-2008% curative-intent (n curative-intent/n total)	B: 2009-2012% curative-intent (n curative-intent/n total)	C: 2013-2020% curative-intent (n curative-intent/n total)
0 (n=1153)	52.1 (148/284)	65.5 (184/281)	71.3 (419/588)
1 (n=4224)	43.9 (374/852)	60.5 (648/1071)	70.7 (1627/2301)
2 (n=3248)	34.8 (165/474)	51.3 (391/762)	67.8 (1365/2012)
3 (n=1328)	15.6 (26/167)	21.8 (76/348)	47.8 (389/813)

**Table 3:** Proportion of patients treated with curative-intent radiotherapy across each stage and time period.

Stage	A: 2005-2008% curative-intent (n curative-intent/n total)	B: 2009-2012% curative-intent (n curative-intent/n total)	C: 2013-2020% curative-intent (n curative-intent/n total)
I (n=2254)	76.9 (247/321)	91.4 (405/443)	97.5 (1453/1490)
II (n=1029)	70.3 (111/158)	84.8 (206/243)	91.6 (575/628)
III (n=3237)	40.4 (223/552)	66.4 (538/810)	75.9 (1424/1875)
IV* (n=2360)	2.11 (3/142)	9.96 (51/512)	14.9 (255/1706)

*Patients with oligometastatic disease treated with curative intent.

**Table 4:** Proportion of patients treated with curative-intent radiotherapy across each PS and time period for stage III patients only.

PS	A: 2005-2008% curative-intent (n curative-intent/n total)	B: 2009-2012% curative-intent (n curative-intent/n total)	C: 2013-2020% curative-intent (n curative-intent/n total)
0 (n=451)	66.7 (48/72)	79.4 (77/97)	87.2 (246/282)
1 (n=1430)	46.0 (116/252)	77.9 (306/393)	85.2 (669/785)
2 (n=819)	28.7 (31/108)	57.6 (110/191)	72.1 (375/520)
3 (n=296)	10.5 (4/38)	32.9 (27/82)	34.1 (60/176)

**Figure 4:**
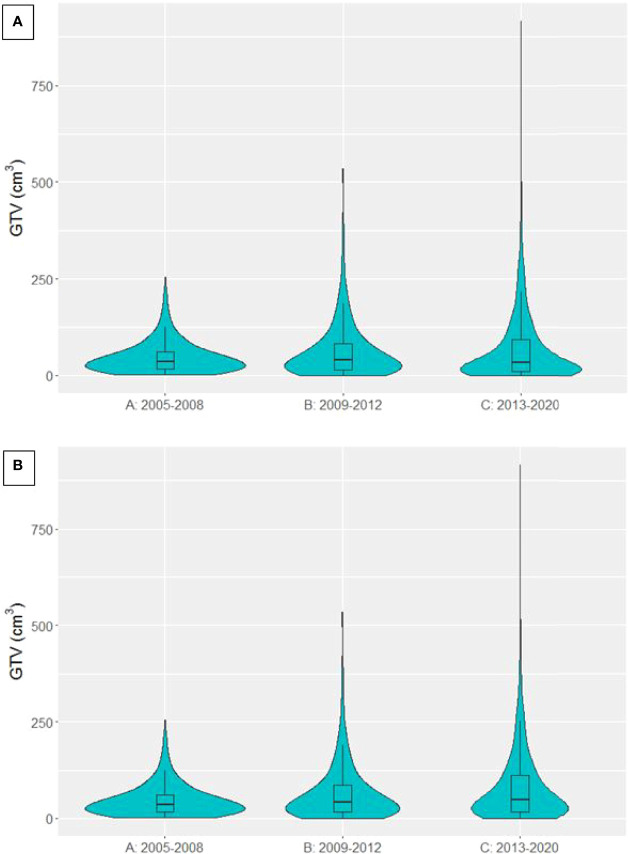
Violin plot presenting the distribution of GTVs in patients treated with curative-intent radiotherapy in each time period. **(A)** SABR patients included **(B)** SABR patients excluded.

GTV data was available for 4306 patients treated with curative-intent. The distribution of GTVs in each time period is presented in **Figure 4A**, showing larger GTVs have been treated in group C compared to A and B. Median GTV was 35.5 cm^3^ [16.8, 60.1], 39.2 cm^3^ [15.1, 82.9] and 32.5 cm^3^ [9.9, 91.8] for groups A, B and C respectively. There was a significant decrease in median GTV between time periods B and C (p=0.00597). However, when patients treated with SABR (n=546) were removed from the analysis (violin plot in **Figure 4B**), median GTV was 35.5 cm^3^ [16.8, 60.1], 41.7 cm^3^ [16.3, 85.8] and 47.6 cm^3^ [17.6, 112.1] for groups A, B and C respectively, showing a significant increase in GTV size in each time period in non-SABR patients (A to B, p=0.00383; B to C, p=0.00136). The maximum treated GTV also increased across each time period, from 254.0 cm^3^ to 534.4 cm^3^ to 916.3 cm^3^.

**Figure 5:**
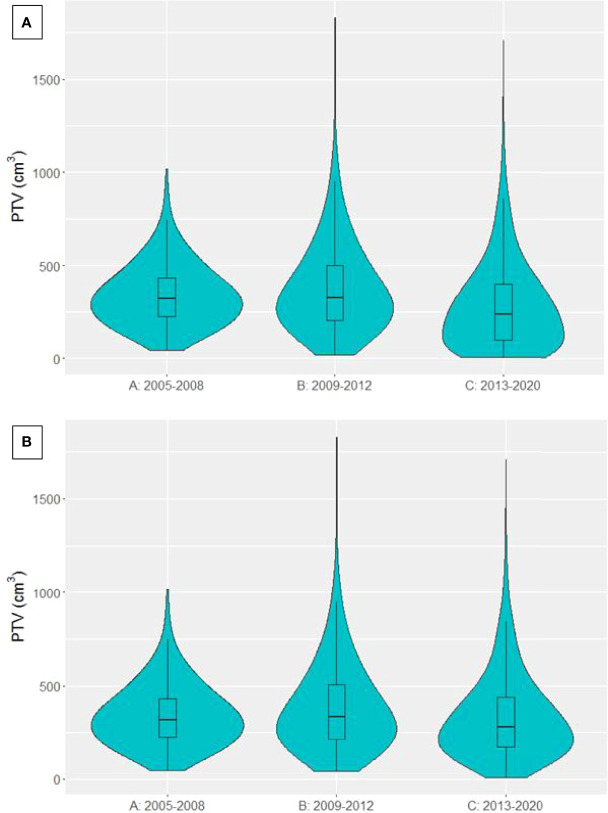
Violin plot presenting the distribution of PTVs from patients treated with curative-intent radiotherapy in each time period. **(A)** SABR patients included **(B)** SABR patients excluded.

PTV data was available for 4915 curative-intent patients. The distribution of PTVs in each time period is presented in **Figure 5**. Median PTV was 319.2 cm^3^ [225.8, 433.2], 326.3 cm^3^ [202.3, 502.2] and 235.9 cm^3^ [97.8, 401.7] for groups A, B and C respectively. There was a significant decrease in PTV between time periods B and C (p<0.0001). When patients treated with SABR were removed from the analysis, median PTV was 319.2 cm^3^ [225.8, 433.2], 334.1 cm^3^ [211.8, 506.7] and 282.2 cm^3^ [169.7, 438.9] for groups A, B and C respectively, again showing a significant decrease in PTV between time periods B and C (p<0.0001).

**Figure 6:**
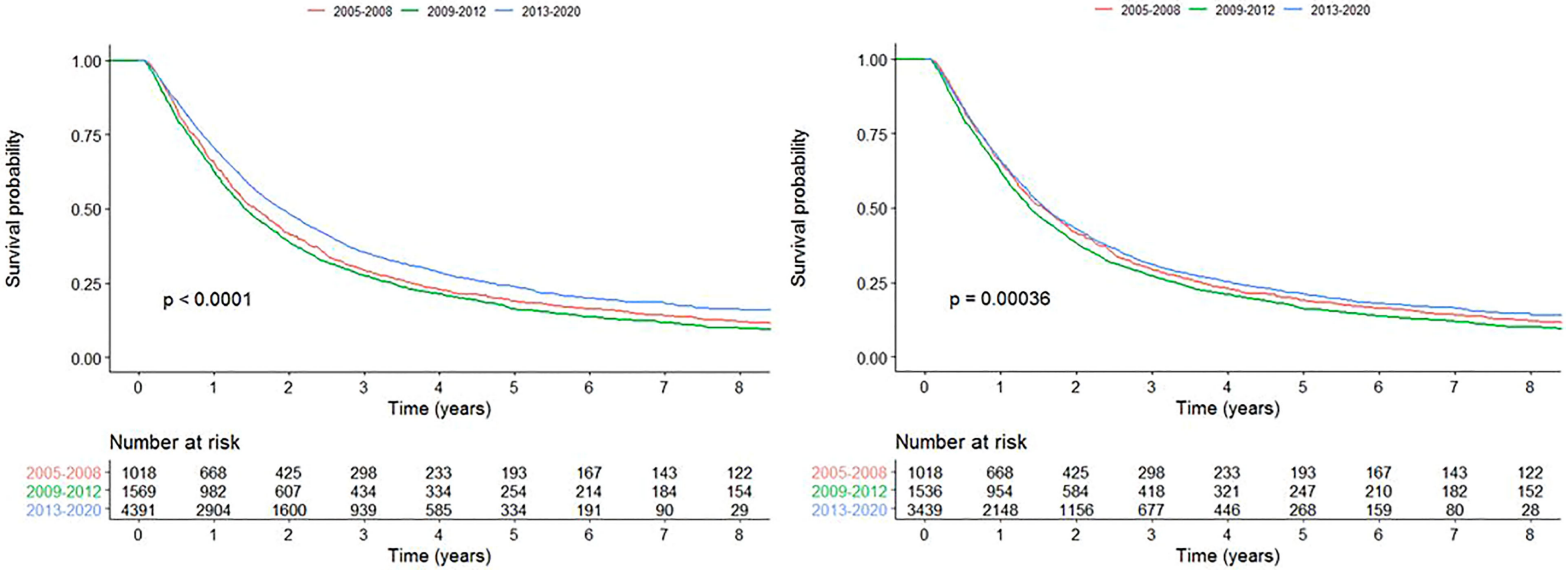
Kaplan-Meier survival curves for each time period for all patients treated with curative-intent radiotherapy (left) and curative-intent without SABR (right).

Univariable survival analysis showed that the survival of patients treated with curative-intent radiotherapy has significantly improved in time period C compared to A (HR=0.847 (0.786-0.913), p<0.001). When patients treated with SABR were removed from the analysis, there was only a survival benefit for patients in time period B compared to A (HR=1.09 (1.00-1.18), p=0.0486), not for time period C compared to A (HR=0.949 (0.879-1.02), p=0.180). Kaplan-Meier curves are presented in **Figure 6** for all curative-intent patients and curative-intent without SABR. Multivariable survival analysis, however, showed a survival benefit for patients treated in time period C compared to A for all curative-intent patients (HR=0.725 (0.632-0.831), p<0.001) as well as when patients treated with SABR were removed from the analysis (HR=0.757 (0.658-0.870), p<0.001). Full results are presented in **Supplementary Tables 3, 4**.

**Figure 7:**
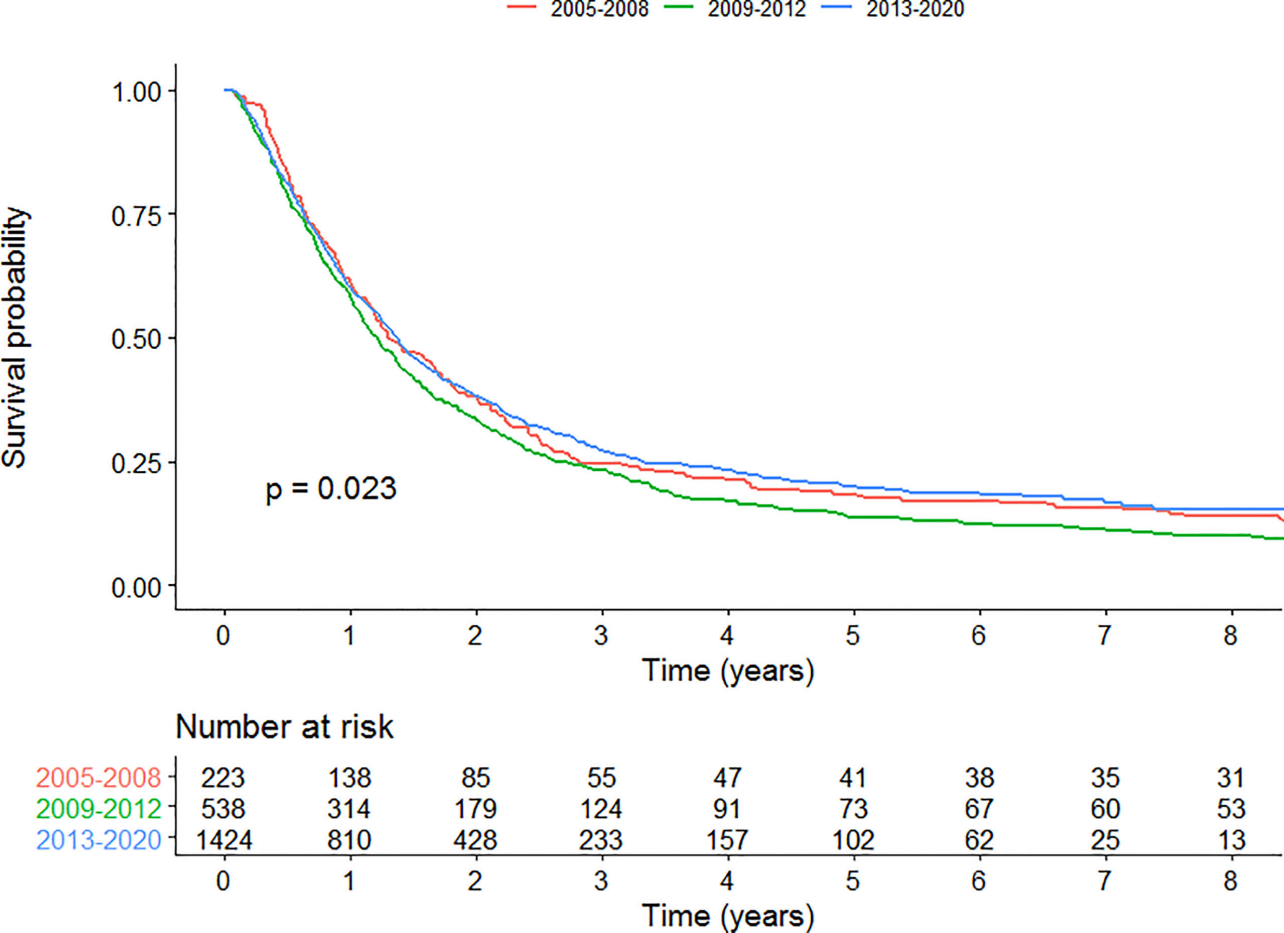
Kaplan-Meier survival curves for each time period for patients with stage III disease curative-intent radiotherapy.

We conducted an analysis in patients with stage III disease. Kaplan-Meier curve is presented in **Figure 7** for patients with stage III treated with curative-intent. Univariable survival analysis showed no significant improvement or worsening of survival for time period C compared to A (HR=0.969 (0.832, 1.13), p=0.683). Multivariable survival analysis however, showed a survival benefit for patients treated in time period C compared to A for patients with stage III disease treated with curative-intent (HR=0.740 (0.600-0.913), p=0.00489). Full results are presented in **Supplementary Table 5**.

## Discussion

In this big data analysis, there has been a steady increase in the proportion of patients treated with curative-intent radiotherapy, across all PS groups and stages of disease. In addition, survival improved in the era when there was full access to IMRT (2013-2020) compared to no access to IMRT (2005-2008) when clinical variables were adjusted for. The introduction of IMRT has allowed the delivery of curative-intent doses to patients with tumours previously considered to be unsuitable for such an approach due to large volume or proximity to critical organs at risk. In addition, the normal tissue sparing that IMRT facilitates enabled the treatment of patients with poorer performance status due to better tolerance of the treatment.

Our analysis showed that the proportion of patients with stage III lung cancer receiving curative-intent treatment has increased over the time periods, across all PS. This change has been partly facilitated by IMRT which allows the treatment of large and complex volumes. Other factors may have played a role, such as 4D CT planning (introduced 2011) facilitating more individualised treatment volumes, and also availability of radiotherapy at new satellite centres from 2010, allowing more patients (particularly the elderly and patients with poorer PS) to be treated nearer home. Accepting this, we still feel that as GTV volumes increased over the time periods studied, the introduction of IMRT is likely to have contributed greatly towards the proportion of patients able to receive curative-intent treatment. Baseline PET imaging has been standard at our institution since 2001 and so should not account for differences observed between the groups.

The survival benefit demonstrated on multivariable analysis was not seen in the unadjusted analysis, reflecting that patients with poorer performance status and larger tumours are being treated in the latest time period. As lung cancer outcome is associated with tumour volume (17, 18), it was expected that the survival in this group might have been worse in comparison to earlier time frames. However, survival improved for patients in the latest time period despite larger gross tumour volumes and an increased number of patients with poorer PS suggesting that planning with IMRT leads to at least non-inferior survival. In particular, when patients treated with SABR were removed from the analysis we showed that despite a significant increase in GTV in patients treated with curative-intent, the survival benefit in the latest time period remained.

Whilst this survival gain could be partly attributed to IMRT it is important to recognise that other changes in lung cancer management have occurred in the intervening time period we examined, and so we cannot claim that IMRT has directly led to an improvement in survival. Technological advances such as SABR, 4D radiotherapy and image guidance radiotherapy have allowed reduced radiotherapy planning margins, leading to reduced normal tissue doses. The doses we used for curative intent stayed the same throughout the study. In our series, median PTV volumes were lower in the later timeframe (C) compared to either of the earlier timeframes, even when patients treated with SABR were excluded. This is likely to reflect a change in our CTV-PTV expansion margins which were introduced in the later time period following a move to daily image verification. It is unlikely that this reduction in PTV volume is responsible for the increased survival seen in the later timeframe, as although the difference was found to be statistically significant, in clinical terms the differences in PTV volume seen between group C and groups A and B is small. Also, GTV volume is known to be an independent prognostic factor for lung cancer survival, and we have previously demonstrated that this parameter increased between the three time periods.

Non-radiotherapy factors such as improved diagnostic imaging techniques, endobronchial ultrasound (EBUS) and associated stage migration, a change in staging classification, improvements in systemic therapy and supportive care may all have led to better outcomes. With regards to systemic therapy, the last 15 years have seen better integration of radiotherapy and systemic treatment, as well as the development of more targeted agents and immunotherapy that can be used on progression. Unfortunately due to the fact that this study started in 2005 data on chemotherapy were not as complete in the first time period due to lack of availability of electronic records for systemic therapy at the time. It was therefore not possible to guarantee a full, accurate and therefore meaningful collection of data on systemic treatment the patient may have received at the time of radiotherapy, or subsequently on progression. It is worth noting however that systemic treatment in the context of concurrent chemoradiotherapy had not changed significantly until the introduction of adjuvant Durvalumab, which has only been in routine use in the U.K. since 2019 (the latter part of our latest time period).

There are other limitations to this study including its retrospective design, and as is always the case when performing big data analyses, there is a significant amount of missing data within the clinical variables, including the lack of data on systemic therapy. This was more evident in the earlier time frames which were prior to our in house electronic e-record being created, which facilitated the prospective collection of key data on outcome forms. We feel the large number of patients included in this analysis in part mitigates the issue of missing data (19). Furthermore, this study reports on a unique dataset that evaluates real-world data from patients that are typically excluded from clinical trials. It is also worth noting that we have purposefully included a heterogenous population of lung cancer patients with differing histologies into this analysis as we were interested in evaluating the impact of IMRT on curative-intent treatment. Admittedly the dose threshold for curative intent of greater than 40Gy may also have included patients with NSCLC who did not fully complete their treatment, but in the context of such a large study, the numbers of patients whom this applies to are expected to be low.

These results are of particular importance in the UK, following publication of the most recent national lung cancer audit (10). This highlighted that the majority of stage III NSCLC patients are receiving best supportive care or palliative treatment, even when patients have a PS of 0/1. In addition, there was a large regional variation in the percentage of patients receiving curative intent treatment from 8-80% (10). It has been suggested that the centres offering a greater proportion of patients curative intent treatment may have better access to optimal radiotherapy planning techniques and image guided treatment (20). Indeed, in the Royal College of Radiologists (RCR) published consensus statements for radiotherapy for lung cancer, it is recommended that patients receiving radical radiotherapy are planned with advanced techniques such as IMRT or VMAT (21).

The implementation of IMRT for the curative-intent treatment of lung cancer has lagged behind that of other disease sites such as head& neck cancers. This may stem from a perceived lack of high level evidence for using the technique. To date, there has only been one prospective study looking at the impact of IMRT on treatment toxicity and survival (12). Chun et al. compared the outcome of patients treated with IMRT to 3D-CRT within the RTOG 0617 trial, reporting that despite larger planning target volumes in the IMRT group, patients had a lower rates of grade 3+ pneumonitis and higher cardiac doses, however no difference in survival between the groups was observed (12). A retrospective study by Yom et al. showed that patients treated with IMRT had larger GTVs compared to matched patients treated with 3D-CRT. Similarly to Chun et al., they reported lower rates of grade 3+ pneumonitis in the IMRT group (22). On the other hand, due to the complexity and cost of delivering IMRT, it has been suggested that 3D-CRT is still an equally sound option for locally advanced NSCLC, particularly for less experienced centres (23). A meta-analysis of studies comparing IMRT to 3D-CRT reported survival to be similar between the two techniques, however there were reduced incidence of grade 2 pneumonitis and increased grade 3 oesophagitis in the IMRT group (24). Overall the available data suggests that IMRT facilitates treatment of larger volumes, does not lead to inferior survival in NSCLC patients and should be employed to reduce dose to organs at risk, particularly to the heart and lung (12, 24).

IMRT and other advanced radiotherapy planning techniques offer the opportunity to achieve more than just treating larger volumes. Due to its ability to sculpt dose around the treatment volume, it may be possible to safely deliver a higher dose to the tumour, without compromising normal tissue toxicity. The hypothesis is that higher dose should equate to improved local control, and subsequently better survival. The RTOG 0617 study results however suggested that dose escalating with conventional fractionation does not seem to offer a benefit. It should be noted that only 47% patients in this study were planned with IMRT, dose to the heart was not prioritised in radiotherapy planning and further analysis has shown that higher cardiac dose in this trial is associated with worse survival (12). Since the publication of RTOG 0617, further studies have demonstrated that excess radiation dose to the heart is associated with a decrease in survival (25). A number of studies are have addressed the question of isotoxic dose escalation and dose painting based on FDG PETCT which are facilitated by the use of IMRT (26).

Looking forward, it may be possible in the future to perform causal inference analyses, which would help establish whether the increased proportion of patients treated with curative intent, and their improved survival, is indeed attributable to the introduction on IMRT. The data could also be enhanced by including treatment related toxicity, something that can now be achieved through the use of patient reported outcomes (ePROMS) and proactive, prospective clinician reported toxicity, which we are now documenting at our centre on an eform at each outpatient visit (27).

In summary, this big data analysis has demonstrated that the introduction of IMRT was associated with an increasing proportion of patients with lung cancer receiving curative-intent radiotherapy, across all PS and stages of disease. Despite treating larger, more complex tumours with curative-intent, and more patients with poor performance status, a survival benefit was seen for patients treated when full access to IMRT was available. This study highlights the impact IMRT has had on our practice, acknowledging that other contributing factors such as improvement in staging, technical radiotherapy and systemic therapy may have also contributed to the improved survival. We would recommend that IMRT is available for routine use for lung cancer patients who are being considered for treatment with curative intent. Current evidence suggests that this technique, at the very least, leads to non-inferior outcomes, and may facilitate improved outcomes firstly through the greater number of patients with stage III disease being able to receive a curative-intent dose, and secondly through a reduction of dose to the normal tissues.

## Data Availability Statement

The raw data supporting the conclusions of this article will be made available by the authors, without undue reservation.

## Author Contributions

All authors contributed to the article and approved the submitted version.

## Funding

This work was supported by CRUK *via* the funding to Cancer Research UK Manchester Centre: [C147/A18083] and [C147/A25254]. CF-F is supported by NIHR Manchester Biomedical Research Centre.

## Conflict of Interest

The authors declare that the research was conducted in the absence of any commercial or financial relationships that could be construed as a potential conflict of interest.

## Publisher’s Note

All claims expressed in this article are solely those of the authors and do not necessarily represent those of their affiliated organizations, or those of the publisher, the editors and the reviewers. Any product that may be evaluated in this article, or claim that may be made by its manufacturer, is not guaranteed or endorsed by the publisher.
