# Supplementary material for: Impact of Introducing Intensity Modulated Radiotherapy on Curative Intent Radiotherapy and Survival for Lung Cancer
Source: Front Oncol. 2022 May 31;12:835844. doi: 10.3389/fonc.2022.835844 (PMC9197586; doi:10.3389/fonc.2022.835844)
Supplement: Supplementary file 1 [file DataSheet_1.docx]

**Supplementary materials**

**Supplementary Figure 1** Percentage of patients treated with curative versus palliative intent, non-SABR radiotherapy year on year from 2005 to 2020.


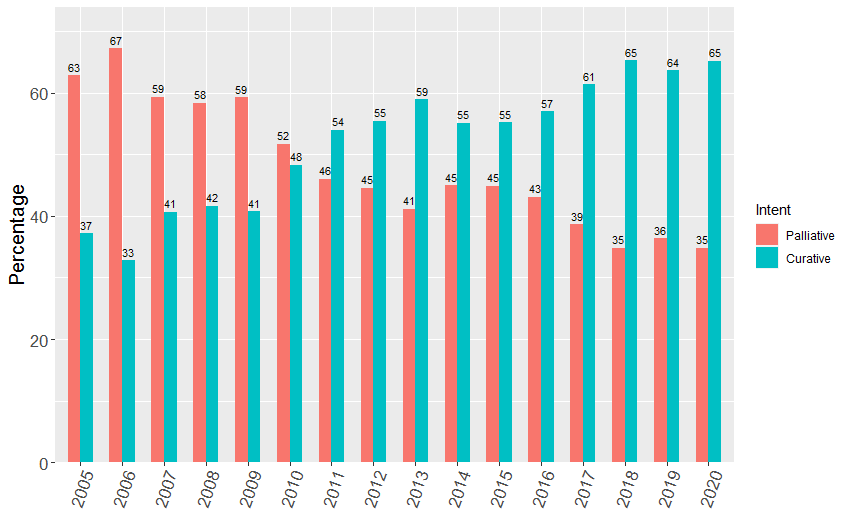


**Supplementary Figure 2** Percentage of patients treated with curative versus palliative intent, non-SABR radiotherapy in each of the pre-specified time periods.


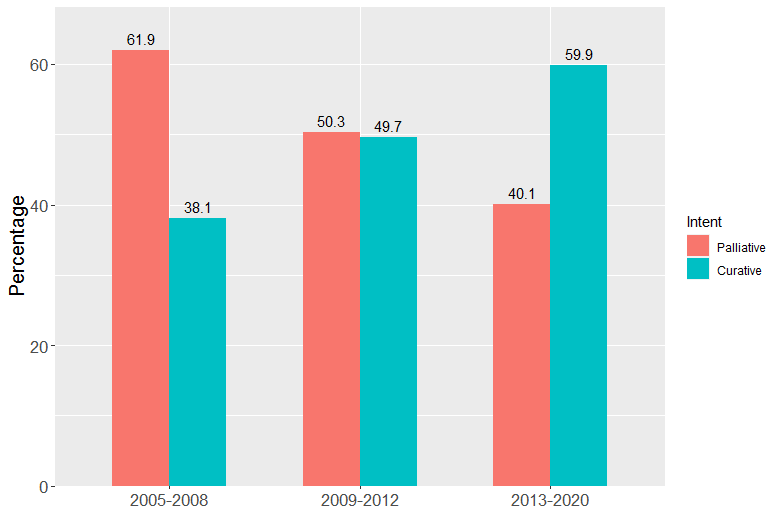


**Supplementary Table 1** Proportion of patients treated with curative-intent, non-SABR radiotherapy across each PS and time period.

| **PS** | **A: 2005-2008**  **% curative-intent**  **(n curative-intent/n PS)** | **B: 2009-2012**  **% curative-intent**  **(n curative-intent/n PS)** | **C: 2013-2020**  **% curative-intent**  **(n curative-intent/n PS)** |
| --- | --- | --- | --- |
| 0  (n=1119) | 52.1  (148/284) | 65.1  (181/278) | 69.7  (388/557) |
| 1  (n=3938) | 43.9  (374/852) | 60.1  (636/1059) | 66.7  (1353/2027) |
| 2  (n=2863) | 34.8  (165/474) | 59.7  (381/752) | 60.5  (990/1637) |
| 3  (n=1190) | 15.6  (26/167) | 21.4  (74/346) | 37.4  (253/677) |

**Supplementary Table 2** Proportion of patients treated with curative-intent, non-SABR radiotherapy across each stage and time period.

| **Stage** | **A: 2005-2008**  **% curative-intent**  **(n curative-intent/n stage)** | **B: 2009-2012**  **% curative-intent**  **(n curative-intent/n stage)** | **C: 2013-2020**  **% curative-intent**  **(n curative-intent/n stage)** |
| --- | --- | --- | --- |
| I  (n=1420) | 76.9  (247/321) | 90.9  (378/416) | 94.6  (646/683) |
| II  (n=1008) | 70.3  (111/158) | 84.8  (206/243) | 91.3  (554/607) |
| III  (n=3231) | 40.4  (223/552) | 66.3  (536/808) | 75.9  (1420/1871) |
| IV  (n=2351) | 2.11  (3/142) | 9.96  (51/512) | 14.5  (246/1697) |

**Supplementary Table 3** Survival analysis results from the multivariable analysis of all curative-intent patients. 3188 patients with no missing variables were included.

|  | **HR (95% CI)_** | ***P* value** |
| --- | --- | --- |
| Time period (ref A: 2005-2008) |  |  |
| B: 2009-2012 | 0.953 (0.822, 1.11) | 0.523 |
| C: 2013-2020 | 0.725 (0.632, 0.831) | <0.001 |
| PS (ref 0) |  |  |
| 1 | 1.31 (1.15, 1.50) | <0.001 |
| 2 | 1.64 (1.42, 1.89) | <0.001 |
| 3 | 1.73 (1.44, 2.08) | <0.001 |
| 4 | 1.28 (0.318, 5.16) | 0.726 |
| Stage (ref I) |  |  |
| II | 1.54 (1.36, 1.74) | <0.001 |
| III | 1.67 (1.50, 1.86) | <0.001 |
| IV | 2.04 (1.70, 2.43) | <0.001 |
| Age at start of treatment (years) | 1.01 (1.01,1.02) | <0.001 |
| GTV (cm^3^) | 1.00 (1.00, 1.00) | <0.001 |

**Supplementary Table 4** Survival analysis results from the multivariable analysis of curative-intent patients without SABR. 2749 patients with no missing variables were included.

|  | **HR (95% CI)_** | ***P* value** |
| --- | --- | --- |
| Time period (ref A: 2005-2008) |  |  |
| B: 2009-2012 | 0.966 (0.832, 1.12) | 0.646 |
| C: 2013-2020 | 0.757 (0.658, 0.870) | <0.001 |
| PS (ref 0) |  |  |
| 1 | 1.29 (1.12, 1.47) | <0.001 |
| 2 | 1.58 (1.37, 1.83) | <0.001 |
| 3 | 1.62 (1.33, 1.97) | <0.001 |
| 4 | 1.04 (0.145, 7.40) | 0.972 |
| Stage (ref I) |  |  |
| II | 1.41 (1.24, 1.61) | <0.001 |
| III | 1.53 (1.37, 1.72) | <0.001 |
| IV | 1.93 (1.59, 2.33) | <0.001 |
| Age at start of treatment (years) | 1.01 (1.01,1.02) | <0.001 |
| GTV (cm^3^) | 1.00 (1.00, 1.00) | <0.001 |

**Supplementary Table 5** Survival analysis results from the multivariable analysis of stage III curative-intent patients. 1370 patients with no missing variables were included.

|  | **HR (95% CI)_** | ***P* value** |
| --- | --- | --- |
| Time period (ref A: 2005-2008) |  |  |
| B: 2009-2012 | 0.966 (0.771, 1.21) | 0.767 |
| C: 2013-2020 | 0.740 (0.600, 0.913) | 0.00489 |
| PS (ref 0) |  |  |
| 1 | 1.30 (1.09, 1.54) | 0.00282 |
| 2 | 1.58 (1.30, 1.91) | <0.001 |
| 3 | 1.86 (1.37, 2.52) | <0.001 |
| Age at start of treatment (years) | 1.01 (1.01,1.02) | <0.001 |
| GTV (cm^3^) | 1.00 (1.00, 1.00) | <0.001 |
